# Aspirin Compared to Low Intensity Anticoagulation in Patients with Non-Valvular Atrial Fibrillation. A Systematic Review and Meta-Analysis

**DOI:** 10.1371/journal.pone.0142222

**Published:** 2015-11-12

**Authors:** Fernando J. Vazquez, Joaquín P. Gonzalez, Esteban Gándara

**Affiliations:** 1 Internal Medicine Research Unit, Internal Medicine Department, Hospital Italiano de Buenos Aires, Buenos Aires, Argentina; 2 Internal Medicine Department, Hospital Italiano de Buenos Aires, Buenos Aires, Argentina; 3 Hospital Universitario, Universidad Nacional de Cuyo, Mendoza, Argentina; 4 Thrombosis Program, Division of Hematology-Department of Medicine, University of Ottawa-Ottawa Hospital, Ottawa, Canada; 5 Ottawa Hospital Research Institute, Ottawa, Canada; University of Perugia, ITALY

## Abstract

**Background:**

Despite its lack of efficacy, aspirin is commonly used for stroke prevention in atrial fibrillation. Since prior studies have suggested a benefit of low-intensity anticoagulation over aspirin in the prevention of vascular events, the aim of this systematic review was to compare the outcomes of patients with non-valvular atrial fibrillation treated with low-intensity anticoagulation with Vitamin K antagonists or aspirin.

**Methods:**

We conducted a systematic review searching Ovid MEDLINE, Embase and the Cochrane Central Register of Controlled Trials, from 1946 to October 14^th^, 2015. Randomized controlled trials were included if they reported the outcomes of patients with non-valvular atrial fibrillation treated with a low-intensity anticoagulation compared to patients treated with aspirin. The primary outcome was a combination of ischemic stroke or systemic embolism. The random-effects model odds ratio was used as the outcome measure.

**Results:**

Our initial search identified 6309relevant articles of which three satisfied our inclusion criteria and were included. Compared to low-intensity anticoagulation, aspirin alone did not reduce the incidence of ischemic stroke or systemic embolism OR 0.94 (95% CI 0.57–1.56), major bleeding OR 1.06 (95% CI 0.42–2.62) or vascular death OR 1.04 (95% CI 0.61–1.75). The use of aspirin was associated with a significant increase in all-cause mortality OR 1.66 (95% CI 1.12–2.48).

**Conclusion:**

In patients with non-valvular atrial fibrillation, aspirin provides no benefits over low-intensity anticoagulation. Furthermore, the use of aspirin appears to be associated with an increased risk in all-cause mortality. Our study provides more evidence against the use aspirin in patients with non-valvular atrial fibrillation.

## Introduction

Despite its lack of efficacy and different recommendations by current guidelines, aspirin is commonly used for stroke prevention in non-valvular atrial fibrillation [1–5] (in Table 1 summarize some of the current recommendations by clinical guidelines). Aspirin is commonly used in patients who are not good candidates for oral anticoagulation with vitamin K antagonist (e.g. prior history of major bleeding, low thromboembolic risk, difficulty maintaining an INR in therapeutic range or patient refusal) [6].

**Table 1:** Current recommendation for the use of aspirin in patients with non-valvular atrial fibrillation.

Guideline	Year	Role of aspirin
American College of Chest Physicians [2]	2012	"For patients with non-rheumatic AF, including those with paroxysmal AF, who are (1) at low risk of stroke…we suggest no therapy rather than antithrombotic therapy, and for patients choosing antithrombotic therapy, we suggest aspirin rather than OAC… (2) at intermediate risk of stroke, we recommend OAC rather than no therapy, and we suggest OAC rather than aspirin… and (3) at high risk of stroke, we recommend oral anticoagulation rather than no therapy or aspirin (+/-clopidogrel)…".
American Heart Association [3]	2014	For patients with non-valvular AF with prior stroke, transient ischemic attack (TIA), or a CHA2DS2-VASc score of 2 or greater, OAC are recommended… For patients with non-valvular AF and a CHA2DS2-VASc score of 1, no antithrombotic therapy or treatment with an oral anticoagulant or aspirin may be considered. (Level of Evidence: C)… For patients with non-valvular AF and a CHA2DS2-VASc score of 0, it is reasonable to omit antithrombotic therapy.
European Society of Cardiology [4]	2012	In patients with a CHA2 DS2 -Vac score ≥1, OAC therapy with: adjusted-dose VKA (INR 2–3), a direct thrombin inhibitor… or an oral factor Xa inhibitor … is recommended, unless contraindicated. When patients refuse the use of any OAC, antiplatelet therapy should be considered, using combination therapy with aspirin 75–100 mg plus clopidogrel 75 mg daily (where there is a low risk of bleeding) or—less effectively—aspirin 75–325 mg daily.
National Institute for Health and Care Excellence [5]	2014	Do not offer aspirin monotherapy solely for stroke prevention to people with AF

OAC: Oral anticoagulation; AF: Atrial Fibrillation

Low-intensity anticoagulant treatment using vitamin K antagonists (e.g. fixed mini-doses) is less effective than moderate-intensity therapy [mean target International Normalized Ratio (INR) 2–3] and not recommended for the prevention of stroke in non-valvular atrial fibrillation [7]. Since prior studies have suggested a mortality benefit of low-intensity anticoagulation with vitamin K antagonists over aspirin for preventing vascular events [8], the aim of this systematic review was to compare the outcomes of patients with non-valvular atrial fibrillation treated with aspirin or with low-intensity anticoagulation with Vitamin K antagonists.

## Methods

We conducted a systematic review searching Ovid MEDLINE, Embase and the Cochrane Central Register of Controlled Trials, from 1946 to May 28th 2014 (S1 Text) and updated during review of the manuscript to include the months between May 2014 and October 2015 (S2 Text). The search was designed with the support of a librarian from the Ottawa Hospital Health Services and was supplemented by hand-search of relevant articles, abstract books from international meetings and published reviews.

Randomized controlled trials were included if they: 1) enrolled of patients with non-valvular atrial fibrillation; 2) One of the study arms included patients treated with a low fixed low dose of a Vitamin K antagonists or targeted an INR of less than 1.6. Studies using a combination of low intensity anticoagulation plus aspirin were excluded from the main analysis given the lack of efficacy associated with this combination [9–10] but information was recorded for a sensitivity analysis; 3) the other arm included patients treated with aspirin alone (less than 325mg daily); and 4) and followed patients for at least 3 months.

### Data Extraction and Quality Assessment

All potentially relevant articles were reviewed in full text to ensure that they satisfied the inclusion criteria. Two reviewers (FV and EG) independently assessed the eligibility of all articles identified in the initial search strategy. A third reviewer adjudicated all discrepancies if needed (JG). The "Cochrane Collaboration’s tool for assessing risk of bias" was used to determine the methodological quality of the selected studies.

### Outcome measure

The primary outcome was a combination of ischemic stroke or systemic embolism. Secondary outcomes were ischemic stroke, systemic embolism, major bleeding, death from a vascular cause and all-cause mortality.

### Data Synthesis and Analysis

The random-effects model OR was used as the primary outcome measure, along with the corresponding 95% confidence intervals (CIs]. The I^2^ statistic was used to quantify heterogeneity among the pooled estimates across studies. An I^2^ value less than 25% was considered low-level heterogeneity, 25% to 50% as moderate-level, and greater than 50% as high-level. The Statistical analysis was performed using Review Manager 5.3 (Copenhagen: The Nordic Cochrane Centre, The Cochrane Collaboration, 2014).

## Results

**Fig 1:**
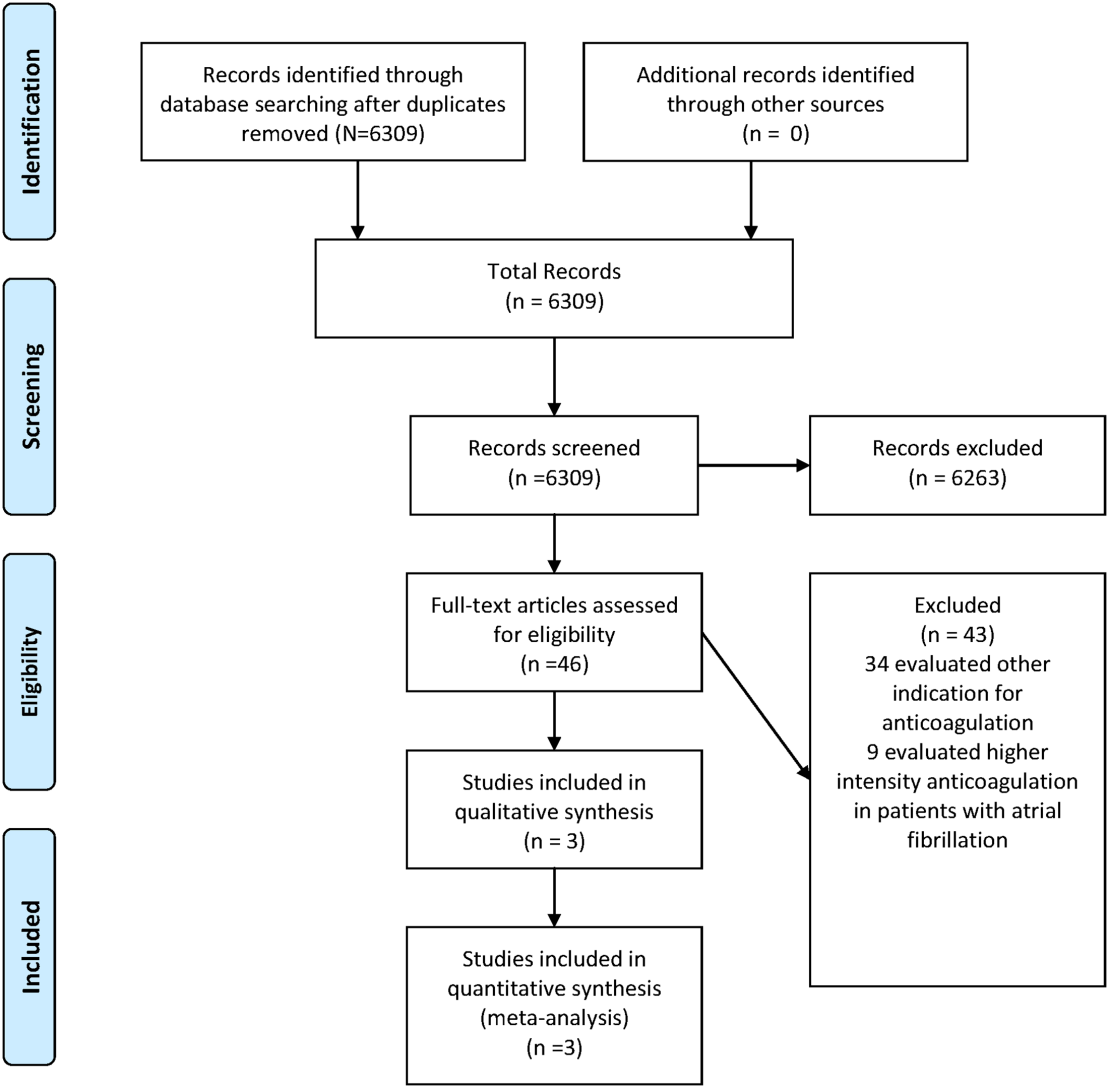
Flow diagram. None of the studies reported a standardized risk assessment score (such as the CHADS-2) to estimate the risk of thromboembolic for their population. The AFASAK II [13] was the only study enrolling patients with a prior history of stroke or transient ischemic attack, and it also included a large number of patients with heart failure (>70%). See Table 1 for studies characteristics and Table 2 for quality assessment. Both the PATAF [11] and Vemmos trial [12] excluded patients with a prior history of stroke or transient ischemic attack. The most common risk factor for thromboembolic event in both trials was hypertension. See Table 2 for studies characteristics and Table 3 for quality assessment.

The meta-analysis was conducted in accordance with the guidelines of the Preferred Reporting Items for Systematic Reviews and Meta-analyses (PRISMA) statement (S1 Table). Our initial search identified 5779 relevant articles until May 28^th^ 2014 and 530relevant articles from the updated search. Of the 6309 articles which 46 were reviewed in full text (S2 Table); of the 46, three satisfied our inclusion criteria and were included (Fig 1). The three trials included were the "Primary Prevention of Arterial Thromboembolism in patients with Non-rheumatic Atrial Fibrillation in Primary Care" (PATAF)[11], Vemmos et al [12], and the "Second Copenhagen Atrial Fibrillation, Aspirin, and Anticoagulation trial" (AFASAK II) [13]. Of the three studies included the PATAF was conducted in Netherlands [11], one in Greece [12], and the AFASAK II in Denmark [13]. One study conducted in Italy was excluded from the analysis as it compared low intensity anticoagulation plus aspirin vs. moderate intensity anticoagulation [14]. The PATAF trial used a fixed dose of warfarin (1.25 mg daily) [11], Vemmos ET used acenocoumarol (1mg daily) [12] and the AFASAK target INR was under1.6 [13]. The doses of aspirin used varied across studies [Vemmos used 100 mg [12], the PATAF used 150 mg [11] and the AFASAK II used 300 mg [13]]. All the studies were open label and in two of them the adjudication of events was done by independent reviewers [11, 13]. The AFASAK II [13] was terminated early following results of other trials, suggesting that of low dose anticoagulation in combination with aspirin was less effective than moderate intensity anticoagulation [15]. The trial conducted by Vemmos et al was terminated early due to an increased risk of bleeding with low dose anticoagulation (less than15 patients per arm) [11].

**Table 2:** Characteristics of the studies included in the main analysis.

Study (year)	Inclusion criteria	Main outcome	Adjudication of events	Intervention	N	Mean Age%	HTN%	Prior stroke/TIA%	DBT%	HF%	Mean INR/TTR	N main outcome
AFASAK II (1998)	Patients 18 years or older with non-valvular chronic AFIB. Patients younger than 60 years with lone AFIB were excluded	Any stroke or a systemic thromboembolic event	End-point committee unaware of treatment status	warfarin 1.25 mg/d	167	74.2	41	5	14	69	1.14 at one month	14
				Aspirin, 300 mg/d	169	73.1	43	8	10	70	—	10
PATAF (1999)	Older than 60 years with chronic AFIB	Stroke (ischemic or haemorrhagic); Systemic arterial; Major haemorrhage; Vascular death	Events were independently reviewed by two members of the event committees	Stratum I: Phenprocoumon or acenocoumarol target INR 1.1–1.6	122	69.4	28	Excluded	8.1	4	1.4/74%	8
				Stratum I: aspirin 150 mg/day	141	70.8	37	Excluded	14	10	—	12
				Stratum II: Phenprocoumon or acenocoumarol target INR 1.1–1.6	157	80.2	77	Excluded	19	2	1.4/74%	37
				Stratum II: Aspirin 150 mg/day	178	80.5	78	Excluded	40	4	—	41
Vemmos (2006)	Patients over 75 years of age with electrocardiographically confirmed chronic or intermittent AFIB within the prior 12 months	Ischemic stroke and systemic embolism	NR	Fixed-dose acenocoumarol 1 mg/day	14	79.9	73	Excluded	12.5	6.3	NR	1
				Aspirin 100 mg/day	15	79.5	71	Excluded	6.7	13		2
Studies not included in the main analysis												
Study (year)	Inclusion criteria	Main outcome	Adjudication of events	Intervention	N	Mean Age%	HTN %	Prior stroke/TIA%	DBT%	HF%	Mean INR/TTR	N reaching main outcome
AFASAK II (1998)	Patients 18 years or older with non-valvular chronic AFIB. Patients younger than 60 years with lone AFIB were excluded	Any stroke or a systemic thromboembolic event	End-point committee unaware of treatment status.	Warfarin 1.25 mg/d plus 300 mg of ASA	171	72.7	39	13	15	74	1.14 at one month	12

AFASAK: Second Copenhagen Atrial Fibrillation, Aspirin, and Anticoagulation;

PATAF: Primary Prevention of Arterial Thromboembolism in patients with Non-rheumatic Atrial Fibrillation in Primary Care;

HTN: Hypertension; DBT: Diabetes; HF: Heart Failure; TTR: Time in therapeutic range

**Table 3:** Quality assessment.

Study	Random sequence generation	Allocation concealment	Blinding of participants/personnel	Blinding of outcome assessment	Incomplete outcome data	Selective reporting
AFASAK	Low risk	Low risk	High risk	Low risk	Low risk	Low risk
PATAF	Low risk	Low risk	High risk	Low risk	Low risk	Low risk
VEMMOS	Unclear risk	Low risk	High risk	High risk	Low risk	Low risk

AFASAK: Second Copenhagen Atrial Fibrillation, Aspirin, and Anticoagulation; PATAF: Primary Prevention of Arterial Thromboembolism in patients with Non-rheumatic Atrial Fibrillation in Primary Care

**Fig 2:**
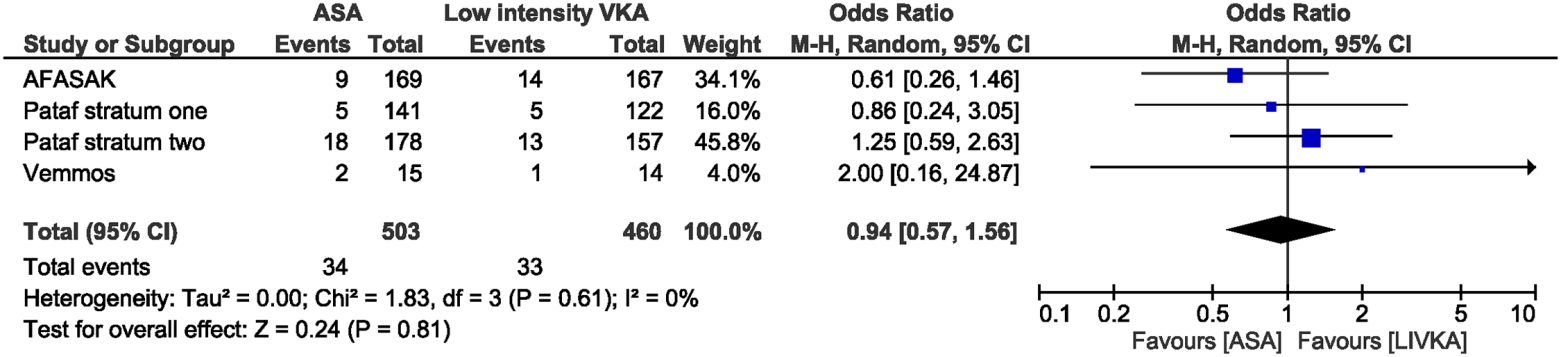
Meta-analysis of ischemic stroke or systemic embolism. There was no difference in the rate of major bleeding [OR 1.06 (95% CI 0.42–2.62); I^2^ 0%] or vascular death [OR 1.04 (95% CI 0.61–1.75); I^2^ 1%] but patients treated with aspirin had an increased risk in all-cause mortality [OR 1.66 (95% CI 1.12–2.48); I^2^ 0%] (Fig 3). The difference in all-cause mortality was driven by an increased risk in non-vascular death in patients treated with aspirin [OR 3.20(95% CI 1.31–7.82); I^2^ 0%], whereas the risk for death from unknown causes not significantly different [OR 1.525 (95% CI 0.65–3.55; I^2^ 0%]. Table 4 provides the number of events in each study.

In total 460 patients treated with low-intensity anticoagulation and 503 treated with aspirin were included in the analysis. Compared to low-intensity anticoagulation, aspirin did not reduce the incidence of ischemic stroke and systemic embolism [OR 0.94 (95% CI 0.57–1.56); I^2^ 0%] (Fig 2), ischemic stroke [OR 0.85 (95% CI 0.49–1.48); I^2^ 0%], systemic embolism [OR 1.39 (95% CI 0.33–5.9); I^2^ 6%].

**Table 4:** Event rates in individual studies.

Study	N	Stroke or embolism	ICH	Stroke	Death	Vascular death	Embolism	Bleeding	N	Stroke or embolism	ICH	Stroke	Death	Vascular death	Embolism	Bleeding
		**n/%**	**n/%**	**n/%**	**n/%**	**n/%**	**n/%**	**n/%**		**n/%**	**n/%**	**n/%**	**n/%**	**n/%**	**n/%**	**n/%**
	Low intensity anticoagulation								Aspirin							
AFASAK II	167	14/8.3	1/0.5	13/7.7	6/3.4	2/1.1	1/0.5	3/1.7	169	9/5.3	1/0.5	8/4.7	14/8.2	4/2.3	1/0.5	5/2.9
PATAF stratum I	122	42128	1/0.8	3/2.4	8/6.4	4/3.2	2/1.6	1/0.8	141	5/3.5	0	4/2.8	42355	6/4.2	1/0.7	0
PATAF stratum II	157	13/7.8	2/1.2	12/7.6	33/19.8	24/15.2	1/0.6	4/2.4	187	18/9.5	4/2.1	13/6.8	49/25.9	25/13.2	5/2.6	5/2.6
Vemmos	14	1/7.1	0	1/7.1	0	0	0	1/7.1	15	2/13.3	0	2/13.3	0	0	0	0

AFASAK: Second Copenhagen Atrial Fibrillation, Aspirin, and Anticoagulation; PATAF: Primary Prevention of Arterial Thromboembolism in patients with Non-rheumatic Atrial Fibrillation in Primary Care. ICH: Intracranial bleeding

**Fig 3:**
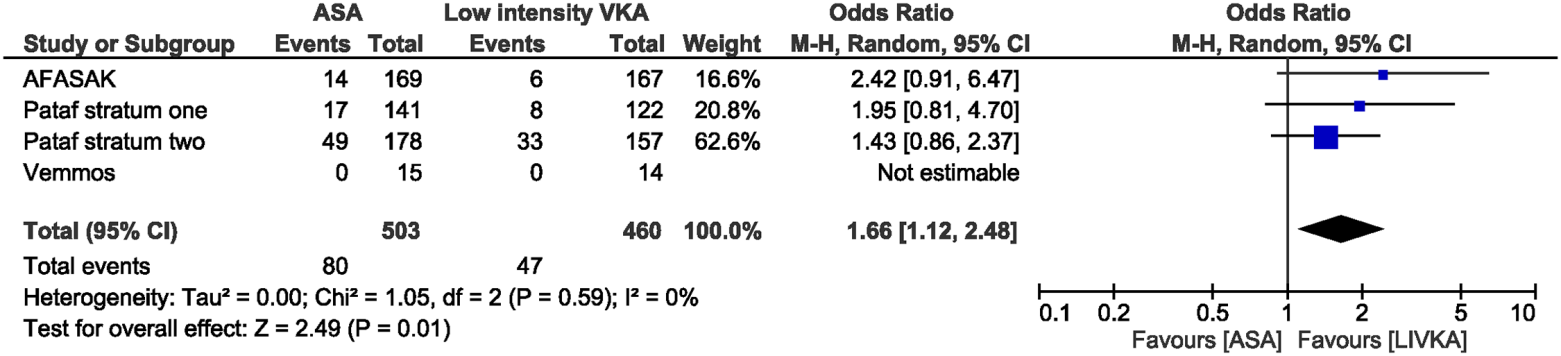
All cause mortality meta-analysis. The addition of a study arm from the AFASAK study [13] comparing aspirin vs. low-intensity anticoagulation plus aspirin did not modify any of the estimates including the reduction in all-cause mortality [OR 1.66(95% CI 1.15–2.38); I^2^ 0%]. Table 3 presents a summary of the number of individual events from each study.

## Conclusions

Compared to low intensity anticoagulation, aspirin does not reduce the incidence of systemic embolism or stroke in patients with non-valvular atrial fibrillation. A novel finding of our meta-analysis was that the use of aspirin was associated with an increased risk in all-cause mortality (driven by an increased risk of non-vascular death). Compared to placebo the use of aspirin in patients with non-valvular atrial fibrillation leads to a modest reduction in the incidence of stroke when [16;17]without an effect all-cause mortality. When moderate intensity anticoagulation (target INR greater than 2) has been compared with aspirin, moderate intensity anticoagulation was associated with a reduction in stroke but not in all-cause mortality [12]. This paradoxical effect leading to a reduction in all-cause mortality of patients treated with low intensity anticoagulation (alone or in combination with antiplatelet agents), has been suggested in studies of primary prevention of cardiovascular events using vitamin K antagonists or direct oral anticoagulants [3;18]. Low intensity anticoagulation has minimal effects on the coagulation cascade anddoes not reduce the levels of important coagulation markers such D-dimer and fibrinogen [19], explaining its ineffectiveness for stroke prevention [20]. Whereas this mild effect in the coagulation cascade is responsible for the mortality will remain hypothesis generating, as the low number of events occurring in each group does not allow to clarify if the events were associated to fatal bleeding or death from other causes.

What are the clinical implications of our findings? Despite not reducing stroke/systemic embolism or mortality, aspirin continues to be commonly used in patients with non-valvular atrial fibrillation [21;22]. In this systematic review we found that not only aspirin does not reduce the incidence of stroke in patients with atrial fibrillation when compared to least effective anticoagulation strategy using vitamin K antagonists, but was associated with a significant increase in mortality. By specifically comparing aspirin to the least effective anticoagulation strategy [7; 23; 24], our results reinforce current recommendations suggesting not to use aspirin in patients for the prevention of stroke in patients with non-valvular atrial fibrillation [1]. An INR under 1.5 was associated with a five-fold increase in the risk of having a stroke in patients taking vitamin K antagonist [23; 24] antagonists. Furthermore, in patients who are not candidates for vitamin K antagonists, the results of the Averroes trial have suggested that apixaban 5 mg twice a day significantly reduced the incidence of stroke without an increase in the risk of major bleeding when compared to aspirin alone [6].

Our systematic review has limitations. First, most of the studies included were terminated before completion, which reduces the power to detect meaningful differences. Second, most of the studies included a low risk population with non-valvular atrial fibrillation. Since the two largest studies were conducted prior to the development of the CHADS-2 score, it is difficult to provide a unified estimate of the risk of stroke across the populations. Third, all but one of the studies were conducted more than 20 years ago and new drugs have shown benefits over vitamin K antagonists and aspirin in patients with non-valvular atrial fibrillation [2]. Fourth, we could not investigate in depth the reasons for the difference in all-cause mortality, an individual patient meta-analysis could have potentiality help to address this issue but the cause of death was unknown in 43% of patients who died in the PATAF trial [7] and 21% of the patients who died in AFASAK [9].

In patients with non-valvular atrial fibrillation, aspirin is not superior to low intensity anticoagulation with vitamin K antagonists. Furthermore, the use of aspirin appears to be associated with an increased risk in all-cause mortality. Our study provides more evidence against the use of aspirin in patients with non-valvular atrial fibrillation.

## Supporting Information

S1 Table 2009 PRISMA checklist. (DOC)

S2 Table Excluded studies. (DOCX)

S1 Text Literature search. (DOCX)

S2 Text Updated literature search. (DOC)
