# Supplementary material for: Aspirin Compared to Low Intensity Anticoagulation in Patients with Non-Valvular Atrial Fibrillation. A Systematic Review and Meta-Analysis
Source: PLoS One. 2015 Nov 12;10(11):e0142222. doi: 10.1371/journal.pone.0142222 (PMC4642960; doi:10.1371/journal.pone.0142222)
Supplement: S2 Table — (DOCX) [file pone.0142222.s002.docx]

S2 Table. Excluded studies

| **Trial** | **Reason for exclusion** | **Reference** |
| --- | --- | --- |
| **Abdul-Rahman** | Included patients with coronary artery by-pass | *Abdul-Rahman I.S., Al-Howaish A.K. Warfarin versus aspirin in preventing tunneled hemodialysis catheter thrombosis: A prospective randomized study. Hong Kong Journal of Nephrology 2007; 9 (1): 23-30.* |
| **AFASAK I** | Atrial fibrillation but target INR >1.6 | *Petersen P, Boysen G, Godtfredsen J, et al. Placebo-controlled, randomised trial of warfarin and aspirin for prevention of thromboembolic complications in chronic atrial fibrillation. The Copenhagen AFASAK study. Lancet 1989;1:175-179* |
| **After** | Included patients with acute coronary syndrome | *Julian DG, Chamberlain DA, Pocock SJ. A comparison of aspirin and anticoagulation following thrombolysis for myocardial infarction (the AFTER study): a multicentre unblinded randomised clinical trial. BMJ. 1996 Dec 7;313(7070):1429-31.* |
| **ALIWAPAS** | Low dose warfarin plus aspirin in APS | *Cuadrado MJ, Bertolaccini ML, Seed PT, et al. Low-dose aspirin vs low-dose aspirin plus low-intensity warfarin in thromboprophylaxis: a prospective, multicentre, randomized, open, controlled trial in patients positive for antiphospholipid antibodies (ALIWAPAS). Rheumatology (Oxford). 2014 Feb;53(2):275-84.* |
| **APRICOT** | Included patients with acute coronary syndrome | *Meijer A, Verheugt FW, Werter CJ, et al. Aspirin versus coumadin in the prevention of reocclusion and recurrent ischemia after successful thrombolysis: a prospective placebo-controlled angiographic study. Results of the APRICOT Study. Circulation. 1993 May;87(5):1524-30* |
| **Aspect** | Included patients with acute coronary syndrome | *van Es RF, Jonker JJ, Verheugt FW, Deckers JW, Grobbee DE; et al. Aspirin and coumadin after acute coronary syndromes (the ASPECT-2 study): a randomised controlled trial. Lancet. 2002 Jul 13;360(9327):109-13* |
| **ATACS pilot** | Included patients with acute coronary syndrome | *Cohen M, Adams PC, Hawkins L, et al. Usefulness of antithrombotic therapy in resting angina pectoris or non-Q-wave myocardial infarction in preventing death and myocardial infarction (a pilot study from the Antithrombotic Therapy in Acute Coronary Syndromes Study Group). Am J Cardiol 1990 Dec 1;66:1287-92* |
| **BAATAF** | Atrial fibrillation but target INR >1.6 | *The Boston Area Anticoagulation Trial for Atrial Fibrillation Investigators. The effect of low-dose warfarin on the risk of stroke in patients with non-rheumatic atrial fibrillation. N Engl J Med 1990;323:1505-1511* |
| **BAFTA** | Atrial fibrillation but target INR >1.6 | *Mant J, Hobbs FD, Fletcher K, et al. Warfarin versus aspirin for stroke prevention in an elderly community population with atrial fibrillation (the Birmingham Atrial Fibrillation Treatment of the Aged Study, BAFTA): a randomised controlled trial. Lancet. 2007 Aug 11;370(9586):493-503* |
| **BOA** | Included patients with leg by-pass | *Efficacy of oral anticoagulants compared with aspirin after infrainguinal bypass surgery (The Dutch Bypass Oral Anticoagulants or Aspirin Study): a randomised trial. Lancet. 2000 Jan 29;355(9201):346-51* |
| **CABADAS** | Included patients with coronary artery by-pass | *van der Meer J, Hillege HL, et al. Prevention of one-year vein-graft occlusion after aortocoronary-bypass surgery: a comparison of low-dose aspirin, low-dose aspirin  plus dipyridamole, and oral anticoagulants. The CABADAS Research Group of the Interuniversity Cardiology Institute of The Netherlands. Lancet. 1993 Jul 31;342(8866):257-64.* |
| **Chen** | Atrial fibrillation but target INR >1.6 | *Chen KP, Huang CX, Huang DJ, et al. Anticoagulation therapy in Chinese patients with non-valvular atrial fibrillation: a prospective, multi-center, randomized, controlled study. Chin Med J (Engl). 2012 Dec;125(24):4355-60.* |
| **Dinh** | Atrial fibrillation but target INR >1.6 | *Dinh T, Baur LH, Pisters R, et al. Aspirin versus vitamin K antagonist treatment guided by transoesophagealechocardiography in patients with atrial fibrillation: a pilot study. Heart. 2014 Apr;100(7):563-8.* |
| **EAFT** | Atrial fibrillation but target INR >1.6 | *Secondary prevention in non-rheumatic atrial fibrillation after transient ischaemic attack or minor stroke. EAFT (European Atrial Fibrillation Trial) Study Group. Lancet. 1993 Nov 20;342(8882):1255-62* |
| **EPSIM** | Included patients with acute coronary syndrome | *A controlled comparison of aspirin and oral anticoagulants in prevention of death after myocardial infarction. N Engl J Med 1982;307:701-8* |
| **Garde** | Included patients with stroke | *Gårde A, Samuelsson K, Fahlgren H, at al. Treatment after transient ischemic attacks: a comparison between anticoagulant drug and inhibition of platelet aggregation. Stroke. 1983 Sep-Oct;14(5):677-81* |
| **German-Austrian** | Included patients with acute coronary syndrome | *Breddin K, Loew D, Lechner K, et al. The German-Austrian aspirin trial: a comparison of acetylsalicylic acid, placebo and phenprocoumon in secondary prevention of myocardial infarction. On behalf of the German-Austrian Study Group. Circulation. 1980 Dec;62(6 Pt 2):V63-72* |
| **Gherli** | Not a randomized study | *Gherli T, Colli A, Fragnito C, et al. Comparing warfarin with aspirin after biological aortic valve replacement: a prospective study. Circulation. 2004 Aug 3;110(5):496-500.* |
| **Helas** | Included patients with heart failure | *Cokkinos DV, Haralabopoulos GC, Kostis JB, et al. Efficacy of antithrombotic therapy in chronic heart failure: the HELAS study. Eur J Heart Fail. 2006 Jun;8(4):428-32.* |
| **Huynh** | Included patients with acute coronary syndrome | *Huynh T, Théroux P, Bogaty P, et al. Aspirin, warfarin, or the combination for secondary prevention of coronary events in patients with acute coronary syndromes and prior coronary artery bypass surgery. Circulation. 2001 Jun 26;103(25):3069-74. Erratum in: Circulation. 2005 Jan 25:111(3):377* |
| **Jung** | Included patients with post pacemaker insertion | *Jung W, Fehske W, Manz M, et al. Randomized comparison of aspirin and phenprocoumon for prevention of right-sided thromboembolic complications associated with transvenous defibrillation leads. Eur Heart J. 1995 Jul;16(7):986-92.* |
| **Kouvaras** | Included patients with with an apical thrombus | *Kouvaras G, Chronopoulos G, Soufras G, et al. The effects of long-term antithrombotic treatment on left ventricular thrombi in patients after an acute myocardial infarction. Am Heart J. 1990 Jan;119(1):73-8* |
| **Lavitola** | Included patients with valvular atrial fibrillation | *Lavitola Pde L, Sampaio RO, Oliveira WA, et al. Warfarin or aspirin in embolism prevention in patients with mitral valvulopathy and atrial fibrillation. Arq Bras Cardiol. 2010 Dec;95(6):749-55* |
| **Martí-Fàbregas** | Included patients with carotid disease | *Martí-Fàbregas J, Cocho D, Martí-Vilalta JL,et al. Aspirin or anticoagulants in stenosis of the middle cerebral artery: A randomized trial. Cerebrovasc Dis. 2006;22(2-3):162-9* |
| **McEnany** | Included patients with coronary artery by-pass | *McEnany MT, Salzman EW, Mundth ED, et al. The effect of antithrombotic therapy on patency rates of saphenous vein coronary artery bypass grafts. J Thorac Cardiovasc Surg. 1982 Jan;83(1):81-9.* |
| **Medical Research Council’s** | Included patients for primary prevention | *Thrombosis prevention trial: randomised trial of low-intensity oral anticoagulation with warfarin and low-dose aspirin in the primary prevention of ischaemic heart disease in men at increased risk. The Medical Research Council's General Practice Research Framework. Lancet. 1998 Jan 24;351(9098):233-41* |
| **MOHR** | Included patients with stroke | *Mohr JP, Thompson JL, Lazar RM, et al. A comparison of warfarin and aspirin for the prevention of recurrent ischemic stroke. N Engl J Med. 2001 Nov 15;345(20):1444-51.* |
| **Palumbo** | Included patients for prevention of VTE | *Palumbo A, Cavo M, Bringhen S, et al. Aspirin, warfarin, or enoxaparin thromboprophylaxis in patients with multiple myeloma treated with thalidomide: a phase III, open-label, randomized trial. J Clin Oncol. 2011 Mar 10;29(8):986-93.* |
| **Pengo** | Low dose plus aspirin | Pengo V, Zasso A, Barbero F, et al. Effectiveness of fixed minidose warfarin in the prevention of thromboembolism and vascular death in nonrheumatic atrial fibrillation. Am J Cardiol. 1998 15;82:433-7 |
| **Shariat** | Included patients with PFO and stroke | *Shariat A, Yaghoubi E, Farazdaghi M, et al. Comparison of medical treatments in cryptogenic stroke patients with patent foramen ovale: A randomized clinical trial. J Res Med Sci. 2013 Feb;18(2):94-8* |
| **SPAF I** | Atrial fibrillation but target INR >1.6 | *Stroke Prevention in Atrial Fibrillation Study. Final results. Circulation. 1991 Aug;84(2):527-39.* |
| **SPAF II** | Atrial fibrillation but target INR >1.6 | *Warfarin versus aspirin for prevention of thromboembolism in atrial fibrillation: Stroke Prevention in Atrial Fibrillation II Study. Lancet. 1994 Mar 19;343(8899):687-91.* |
| **SPIRIT** | Included patients with stroke | *The Stroke Prevention in Reversible Ischemia Trial (SPIRIT) Study Group. A randomized trial of anticoagulants versus aspirin after cerebral ischemia of presumed arterial origin. Ann Neurol. 1997; 42: 857–865* |
| **Swat** | Included patients with stroke | *Stewart B, Shuaib F, Veloso F. Stroke Prevention with Warfarin or Aspirin Trial (SWAT). Stroke 1998;29:304* |
| **Thorthon** | Included patients with PCI | *Thornton MA, Gruentzig AR, Hollman J, King SB 3rd, Douglas JS. Coumadin and aspirin in prevention of recurrence after transluminal coronary angioplasty: a randomized study. Circulation. 1984 Apr;69(4):721-7* |
| **Warcef** | Included patients with heart failure | *Homma S, Thompson JL, Pullicino PM, et al. Warfarin and aspirin in patients with heart failure and sinus rhythm. N Engl J Med. 2012 May 17;366(20):1859-69.* |
| **Waris** | Included patients with acute coronary syndrome | *Hurlen M, Abdelnoor M, Smith P, et al . Warfarin, aspirin, or both after myocardial infarction. N Engl J Med. 2002 Sep 26;347(13):969-74* |
| **WASDId** | Included patients with carotid disease | *Chimowitz MI, Lynn MJ, Howlett-Smith H, et al. Comparison of warfarin and aspirin for symptomatic intracranial arterial stenosis. N Engl J Med. 2005 Mar 31;352(13):1305-16.* |
| **WaSH** | Included patients with heart failure | *Cleland JG, Findlay I, Jafri S, et al . The Warfarin/Aspirin Study in Heart failure (WASH): a randomized trial comparing antithrombotic strategies for patients with heart failure. Am Heart J. 2004 Jul;148(1):157-64* |
| **Waspo** | Atrial fibrillation but target INR >1.6 | *Rash A, Downes T, Portner R, et al. A randomised controlled trial of warfarin versus aspirin for stroke prevention in octogenarians with atrial fibrillation (WASPO). Age Ageing. 2007 Mar;36(2):151-6* |
| **Watch** | Included patients with heart failure | *Massie BM, Collins JF, Ammon SE, et al. Randomized trial of warfarin, aspirin, and clopidogrel in patients with chronic heart failure: the Warfarin and Antiplatelet Therapy in Chronic Heart Failure (WATCH) trial. Circulation. 2009 Mar 31;119(12):1616-24.* |
| **Weber** | Included patients with coronary artery by-pass | *Weber MA, Hasford J, Taillens C, et al. Low-dose aspirin versus anticoagulants for prevention of coronary graft occlusion. Am J Cardiol. 1990 Dec 15;66(20):1464-8.* |
| **WoA Epic Pilot Trial** | Included patients with tissue valves | *Colli A, Mestres CA, Castella M, et al . Comparing warfarin to aspirin (WoA) after aortic valve replacement with the St. Jude Medical Epic heart valve bioprosthesis: results of the WoA Epic pilot trial. J Heart Valve Dis. 2007 Nov;16(6):667-71* |
