# Supplementary material for: Aspirin Compared to Low Intensity Anticoagulation in Patients with Non-Valvular Atrial Fibrillation. A Systematic Review and Meta-Analysis
Source: PLoS One. 2015 Nov 12;10(11):e0142222. doi: 10.1371/journal.pone.0142222 (PMC4642960; doi:10.1371/journal.pone.0142222)
Supplement: S1 Text — (DOCX) [file pone.0142222.s003.docx]

**S1 Text. Literature Search**

Database: Ovid MEDLINE(R) In-Process & Other Non-Indexed Citations and Ovid MEDLINE(R) <1946 to Present>

Search Strategy: May 28, 2014

--------------------------------------------------------------------------------

1 exp Anticoagulants/ (185048)

2 anticoagulant$.tw. (41669)

3 warfarin.tw. (16007)

4 (vitamin adj3 antagonist$).tw. (2340)

5 vka.tw. (462)

6 exp Vitamin K/ai (1572)

7 (Nicoumalone or phenindione or acenocoumarol$ or Sinthrome or dicoumarol$ or nicoumalone or phenprocoumon or Marcoumar or Marcumar or Falithrom or AVK or bishydroxycoumarin$ or coumarin$ or phenprocoumon$).tw. (10810)

8 or/1-7 (208951)

9 exp Platelet Aggregation Inhibitors/ (90778)

10 (antiplatelet$ or anti-platelet$ or antiaggreg$ or anti-aggreg$ or (platelet$ adj5 inhibit$) or (thrombocyt$ adj5 inhibit$)).tw. (39190)

11 (aspirin$ or ASA or dipyridamol$).tw. (59680)

12 Aspirin/ (38067)

13 Dipyridamole/ (7393)

14 (ticlopidine$ or trapidil).tw. (2597)

15 Ticlopidine/ (7576)

16 Trapidil/ (267)

17 ("acetyl salicylic acid$" or "acetylsalicylic acid" or "acetyl-salicylic acid").tw. (7928)

18 clopidogrel$.tw. (7828)

19 (cilostazol or Pletal).tw. (1114)

20 or/9-19 (143630)

21 8 and 20 (16637)

22 randomized controlled trial.pt. (373734)

23 controlled clinical trial.pt. (88369)

24 randomized.ab. (293839)

25 placebo.ab. (153980)

26 clinical trials as topic/ (169939)

27 randomly.ab. (212796)

28 trial.ti. (126488)

29 or/22-28 (903468)

30 exp animals/ not humans/ (3938734)

31 29 not 30 (833705)

32 21 and 31 (3017)

**33 limit 32 to english language (2626)**

Database: EmbaseClassic+Embase<1947 to 2014 May 28>

Search Strategy:

--------------------------------------------------------------------------------

1 *anticoagulant agent/ (31826)

2 anticoagulant$.tw. (61599)

3 warfarin.tw. (23415)

4 (vitamin adj3 antagonist$).tw. (3687)

5 vka.tw. (1044)

6 *antivitamin K/ (1389)

7 (Nicoumalone or phenindione or acenocoumarol$ or Sinthrome or dicoumarol$ or nicoumalone or phenprocoumon or Marcoumar or Marcumar or Falithrom or AVK or bishydroxycoumarin$ or coumarin$ or phenprocoumon$).tw. (16648)

8 expcoumarin anticoagulant/ (75676)

9 or/1-8 (148633)

10 exp *antithrombocytic agent/ (106122)

11 (antiplatelet$ or anti-platelet$ or antiaggreg$ or anti-aggreg$ or (platelet$ adj5 inhibit$) or (thrombocyt$ adj5 inhibit$)).tw. (54110)

12 (aspirin$ or ASA or dipyridamol$).tw. (127509)

13 acetylsalicylic acid/ (162330)

14 dipyridamole/ (21847)

15 acetylsalicylic acid plus dipyridamole/ (804)

16 ticlopidine/ (12662)

17 trapidil/ (693)

18 ("acetyl salicylic acid$" or "acetylsalicylic acid" or "acetyl-salicylic acid").tw. (11589)

19 clopidogrel$.tw. (13601)

20 (cilostazol or Pletal).tw. (1860)

21 or/10-20 (276354)

22 9 and 21 (33598)

23 random$.tw. (894035)

24 placebo$.mp. (334746)

25 double-blind$.tw. (150080)

26 or/23-25 (1112642)

27 22 and 26 (5531)

**28 limit 27 to english language (5033)**

Database: EBM Reviews - Cochrane Central Register of Controlled Trials <April 2014>

Search Strategy: May 28 2014

--------------------------------------------------------------------------------

1 exp Anticoagulants/ (7441)

2 anticoagulant$.tw. (2117)

3 warfarin.tw. (1631)

4 (vitamin adj3 antagonist$).tw. (156)

5 vka.tw. (42)

6 exp Vitamin K/ai (2)

7 (Nicoumalone or phenindione or acenocoumarol$ or Sinthrome or dicoumarol$ or nicoumalone or phenprocoumon or Marcoumar or Marcumar or Falithrom or AVK or bishydroxycoumarin$ or coumarin$ or phenprocoumon$).tw. (351)

8 or/1-7 (9174)

9 exp Platelet Aggregation Inhibitors/ (7699)

10 (antiplatelet$ or anti-platelet$ or antiaggreg$ or anti-aggreg$ or (platelet$ adj5 inhibit$) or (thrombocyt$ adj5 inhibit$)).tw. (3665)

11 (aspirin$ or ASA or dipyridamol$).tw. (13196)

12 Aspirin/ (4094)

13 Dipyridamole/ (552)

14 (ticlopidine$ or trapidil).tw. (519)

15 Ticlopidine/ (1038)

16 Trapidil/ (25)

17 ("acetyl salicylic acid$" or "acetylsalicylic acid" or "acetyl-salicylic acid").tw. (1222)

18 clopidogrel$.tw. (1338)

19 (cilostazol or Pletal).tw. (294)

20 or/9-19 (19313)

21 8 and 20 (1661)

**22 limit 21 to english language (1299)**
