# Supplementary material for: Aspirin Compared to Low Intensity Anticoagulation in Patients with Non-Valvular Atrial Fibrillation. A Systematic Review and Meta-Analysis
Source: PLoS One. 2015 Nov 12;10(11):e0142222. doi: 10.1371/journal.pone.0142222 (PMC4642960; doi:10.1371/journal.pone.0142222)
Supplement: S2 Text — (DOC) [file pone.0142222.s004.doc]

S2 Text. Updated literature search

Database: Ovid MEDLINE(R) In-Process & Other Non-Indexed Citations and Ovid MEDLINE(R) <1946 to Present>

Search Strategy:

--------------------------------------------------------------------------------

1 exp Anticoagulants/ (186162)

2 anticoagulant$.tw. (45670)

3 warfarin.tw. (17862)

4 (vitamin adj3 antagonist$).tw. (3097)

5 vka.tw. (731)

6 exp Vitamin K/ai (1851)

7 (Nicoumalone or phenindione or acenocoumarol$ or Sinthrome or dicoumarol$ or nicoumalone or phenprocoumon or Marcoumar or Marcumar or Falithrom or AVK or bishydroxycoumarin$ or coumarin$ or phenprocoumon$).tw. (11503)

8 or/1-7 (216766)

9 exp Platelet Aggregation Inhibitors/ (96036)

10 (antiplatelet$ or anti-platelet$ or antiaggreg$ or anti-aggreg$ or (platelet$ adj5 inhibit$) or (thrombocyt$ adj5 inhibit$)).tw. (42738)

11 (aspirin$ or ASA or dipyridamol$).tw. (64772)

12 Aspirin/ (40515)

13 Dipyridamole/ (7544)

14 (ticlopidine$ or trapidil).tw. (2671)

15 Ticlopidine/ (8467)

16 Trapidil/ (272)

17 ("acetyl salicylic acid$" or "acetylsalicylic acid" or "acetyl-salicylic acid").tw. (8376)

18 clopidogrel$.tw. (8940)

19 (cilostazol or Pletal).tw. (1289)

20 or/9-19 (153917)

21 8 and 20 (18077)

22 randomized controlled trial.pt. (413926)

23 controlled clinical trial.pt. (91903)

24 randomized.ab. (335258)

25 placebo.ab. (168714)

26 clinical trials as topic/ (179309)

27 randomly.ab. (241971)

28 trial.ti. (147683)

29 or/22-28 (1002713)

30 exp animals/ not humans/ (4130493)

31 29 not 30 (924818)

32 21 and 31 (3278)

33 limit 32 to english language (2856)

34 ("20140529" or 2014053* or 201406* or 201407* or 201408* or 201409* or 20141*).dc. (651355)

35 2015*.dc. (839339)

36 34 or 35 (1490694)

37 33 and 36 (129)

Database: Embase Classic+Embase <1947 to 2015 October 14>

Search Strategy:

--------------------------------------------------------------------------------

1 *anticoagulant agent/ (34199)

2 anticoagulant$.tw. (69861)

3 warfarin.tw. (27708)

4 (vitamin adj3 antagonist$).tw. (5179)

5 vka.tw. (1714)

6 *antivitamin K/ (1716)

7 (Nicoumalone or phenindione or acenocoumarol$ or Sinthrome or dicoumarol$ or nicoumalone or phenprocoumon or Marcoumar or Marcumar or Falithrom or AVK or bishydroxycoumarin$ or coumarin$ or phenprocoumon$).tw. (18225)

8 exp coumarin anticoagulant/ (83375)

9 or/1-8 (164643)

10 exp *antithrombocytic agent/ (110555)

11 (antiplatelet$ or anti-platelet$ or antiaggreg$ or anti-aggreg$ or (platelet$ adj5 inhibit$) or (thrombocyt$ adj5 inhibit$)).tw. (61327)

12 (aspirin$ or ASA or dipyridamol$).tw. (138949)

13 acetylsalicylic acid/ (174508)

14 dipyridamole/ (22446)

15 acetylsalicylic acid plus dipyridamole/ (851)

16 ticlopidine/ (13385)

17 trapidil/ (699)

18 ("acetyl salicylic acid$" or "acetylsalicylic acid" or "acetyl-salicylic acid").tw. (12375)

19 clopidogrel$.tw. (16239)

20 (cilostazol or Pletal).tw. (2165)

21 or/10-20 (300249)

22 9 and 21 (37187)

23 random$.tw. (1041331)

24 placebo$.mp. (366218)

25 double-blind$.tw. (165532)

26 or/23-25 (1272286)

27 22 and 26 (6020)

28 limit 27 to english language (5497)

29 (2014053* or 201406* or 201407* or 201408* or 201409* or 20141*).dd. (787833)

30 2015*.dd. (1603938)

31 29 or 30 (2391771)

32 28 and 31 (478)

33 remove duplicates from 32 (470)

Database: EBM Reviews - Cochrane Central Register of Controlled Trials <September 2015>

Search Strategy:

--------------------------------------------------------------------------------

1 exp Anticoagulants/ (7532)

2 anticoagulant$.tw. (2465)

3 warfarin.tw. (1953)

4 (vitamin adj3 antagonist$).tw. (230)

5 vka.tw. (87)

6 exp Vitamin K/ai (19)

7 (Nicoumalone or phenindione or acenocoumarol$ or Sinthrome or dicoumarol$ or nicoumalone or phenprocoumon or Marcoumar or Marcumar or Falithrom or AVK or bishydroxycoumarin$ or coumarin$ or phenprocoumon$).tw. (369)

8 or/1-7 (9868)

9 exp Platelet Aggregation Inhibitors/ (7778)

10 (antiplatelet$ or anti-platelet$ or antiaggreg$ or anti-aggreg$ or (platelet$ adj5 inhibit$) or (thrombocyt$ adj5 inhibit$)).tw. (4347)

11 (aspirin$ or ASA or dipyridamol$).tw. (15326)

12 Aspirin/ (4133)

13 Dipyridamole/ (552)

14 (ticlopidine$ or trapidil).tw. (538)

15 Ticlopidine/ (1065)

16 Trapidil/ (25)

17 ("acetyl salicylic acid$" or "acetylsalicylic acid" or "acetyl-salicylic acid").tw. (1286)

18 clopidogrel$.tw. (1778)

19 (cilostazol or Pletal).tw. (377)

20 or/9-19 (21993)

21 8 and 20 (1812)

22 randomized controlled trial.pt. (354917)

23 controlled clinical trial.pt. (84752)

24 randomized.ab. (240393)

25 placebo.ab. (137657)

26 clinical trials as topic/ (33071)

27 randomly.ab. (120428)

28 trial.ti. (145878)

29 or/22-28 (607878)

30 exp animals/ not humans/ (4)

31 29 not 30 (607876)

32 21 and 31 (1609)

33 limit 32 to english language (1376)

34 limit 33 to yr="2014 - 2015" (54)
